# Supplementary material for: Bioreactor virome metagenomics sequencing using DNA spike-ins
Source: PeerJ. 2018 Feb 7;6:e4351. doi: 10.7717/peerj.4351 (PMC5807891; doi:10.7717/peerj.4351)
Supplement: Supplemental Information 1 — For each sample is shown from top to bottom: the graphs, final peak statistics and R-code. The statistics show the mean GC content for a peak (mu), the SD of the peak (sigma) and proportion of each peak of the total graph (pi). In the graph, red triangles depicts mu, the red lines are the individuals peaks, the green line is the combined peak and in blue is the original histogram. [file peerj-06-4351-s001.pdf]

Supplement 1: Peak identification using R-package mixdist. For each sample is shown from top to bottom: the graphs, final peak statistics and R-code. The statistics show the mean GC content for a peak (mu), the SD of the peak (sigma) and proportion of each peak of the total graph (pi). In the graph, ▲ depicts mu, the red lines are the individuals peaks, the green line is the combined peak and in blue is the original histogram

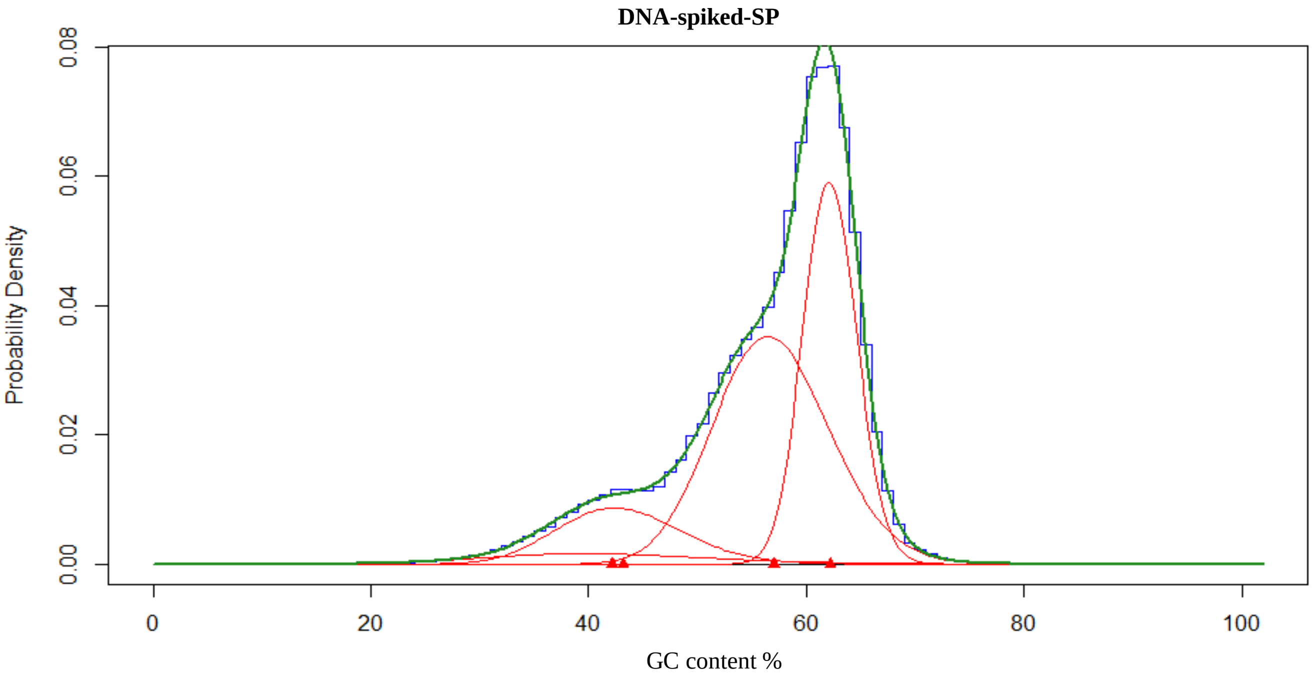

Statistics:

Parameters:

	pi	mu	sigma
1	0.041	42.14	10.31
2	0.124	43.15	5.73
3	0.470	57.00	5.34
4	0.366	62.20	2.47

Standard Errors:

	pi.se	mu.se	sigma.se
1	0.333	29.126	13.351
2	0.449	12.419	12.124
3	0.204	4.183	1.558
4	0.202	0.850	0.811

Analysis of Variance Table

	Df	Chisq	Pr(>Chisq)
Residuals	58	0.1012	1

R-code:

```
d <- as.mixdata(d)
plot(d)
fitd1 <-mix(d,mixparam(c(41,42,53,62),8),"gamma",mixconstr(consigma="NONE"))
summary(fitd1)
plot(fitd1)
fitd2 <- mix(d,coef(fitd1),"gamma")
fitd2 <- mix(d,coef(fitd1),"gamma",iterlim=150)
summary(fitd2)
plot(fitd2)
```

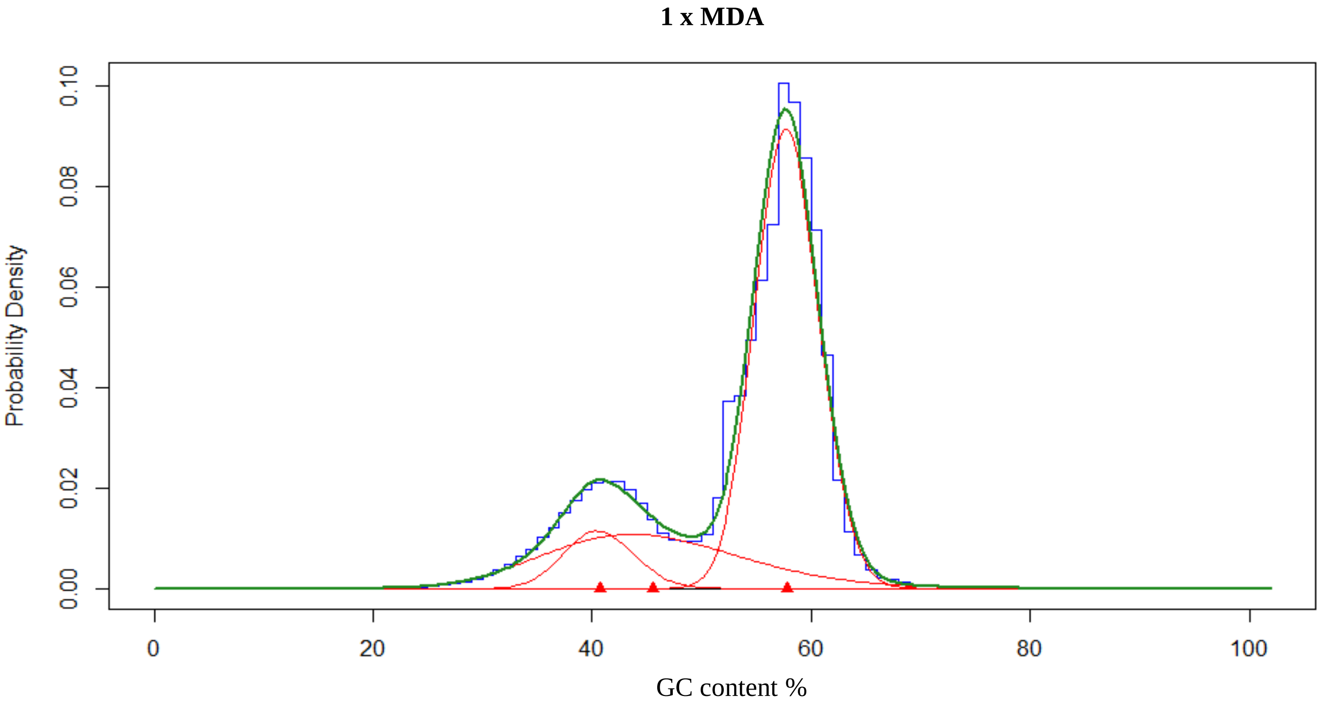

Statistics:

Parameters:

	pi	mu	sigma
1	0.09132	40.71	3.192
2	0.24085	45.53	9.064
3	0.66783	57.83	2.913

Standard Errors:

	pi.se	mu.se	sigma.se
1	0.13103	2.3773	2.976
2	0.17757	3.4903	2.1398
3	0.07988	0.4525	0.4042

Analysis of Variance Table

	Df	Chisq	Pr(>Chisq)
Residuals	62	2.1794	1

R-code:

```
d <- as.mixdata(d)
plot(d)
fitd1 <-mix(d,mixparam(c(41,42,53,62),8),"gamma",mixconstr(consigma="NONE"))
summary(fitd1)
plot(fitd1)
fitd2 <- mix(d,coef(fitd1),"gamma")
fitd2 <- mix(d,coef(fitd1),"gamma",iterlim=150)
summary(fitd2)
plot(fitd2)
```

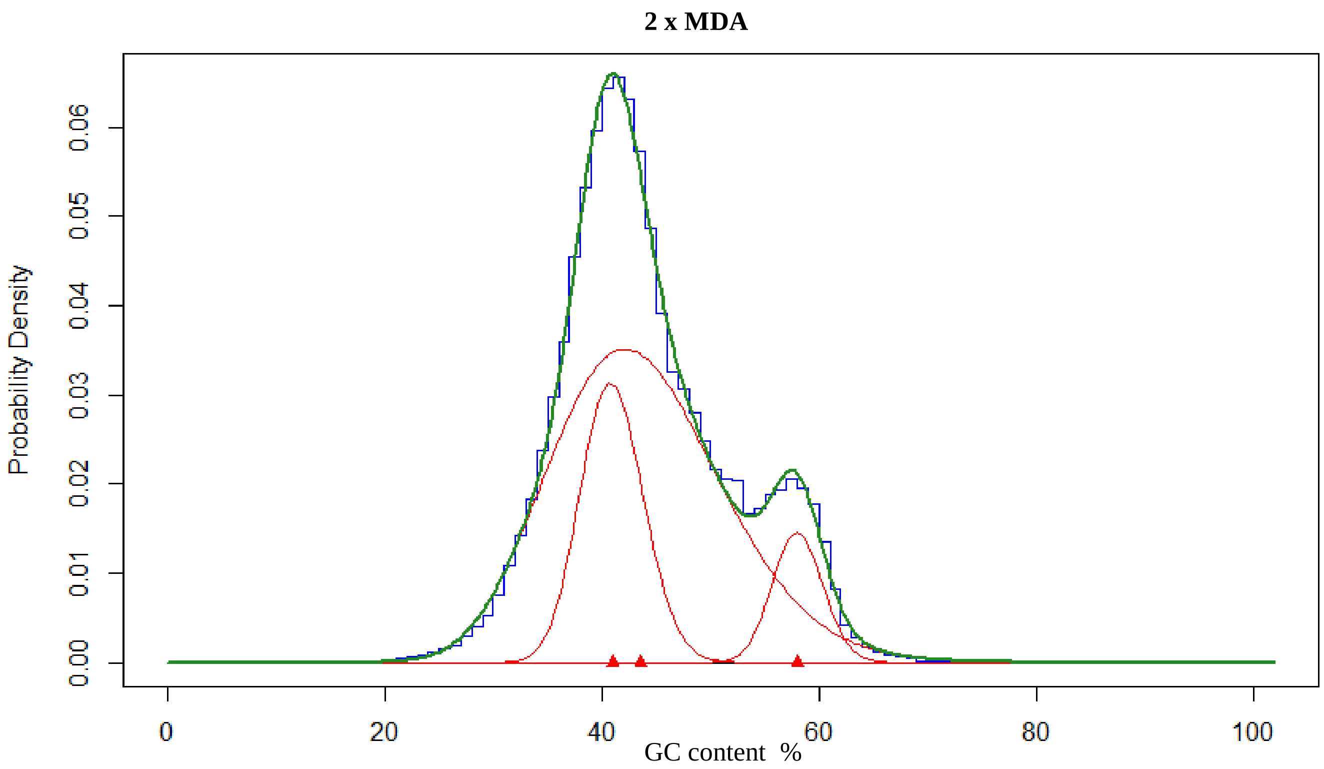

Statistics:

Parameters:

	pi	mu	sigma
1	0.2277	40.98	2.907
2	0.6877	43.58	7.917
3	0.0846	58.05	2.325

Standard Errors:

	pi.se	mu.se	sigma.se
1	0.2138	1.429	1.865
2	0.2294	1.649	1.251
3	0.0611	1.639	1.5

Analysis of Variance Table

	Df	Chisq	Pr(>Chisq)
Residuals	58	1.0566	1

R-code:

```
d <- as.mixdata(d)
plot(d)
fitd1 <-mix(d,mixparam(c(41,42,53,62),8),"gamma",mixconstr(consigma="NONE"))
summary(fitd1)
plot(fitd1)
fitd2 <- mix(d,coef(fitd1),"gamma")
fitd2 <- mix(d,coef(fitd1),"gamma",iterlim=150)
summary(fitd2)
plot(fitd2)
```
