# Supplementary material for: Bioreactor virome metagenomics sequencing using DNA spike-ins
Source: PeerJ. 2018 Feb 7;6:e4351. doi: 10.7717/peerj.4351 (PMC5807891; doi:10.7717/peerj.4351)
Supplement: Supplemental Information 2 — Pre-shearing for Spiked-DNA-GP: 5 microliter was chosen because there was little difference between 5 and 6 microliter at 400 bp, although the shearing of 6 microliter was better. However 5 microliter means less 16S ribosomal DNA and more DNA from the actual sample. (top graph and table). [file peerj-06-4351-s002.pdf]

Supplement 2: Shearing patterns during Library preparation

Pre-shearing for Spiked-DNA-GP: 5 microliter was chosen because there was little difference between 5 and 6 microliter at 400 bp, although the shearing of 6 microliter was better. However 5 microliter means less 16S ribosomal DNA and more DNA from the actual sample. (top graph and table)

Bottom table: The amounts of DNA according to Qubit and Bioanalyzer measurements. The Qubit measurement of 16S ribosomal amplicon was 10 ng/microliter. For the Qubit, this was multiplied by the amount of microliters used i.e. (10 ng/microl * volume). The Bioanalyzer samples were dissolved in 50 microliters, which was multiplied by the concentration in pg/microliter (conc*50).

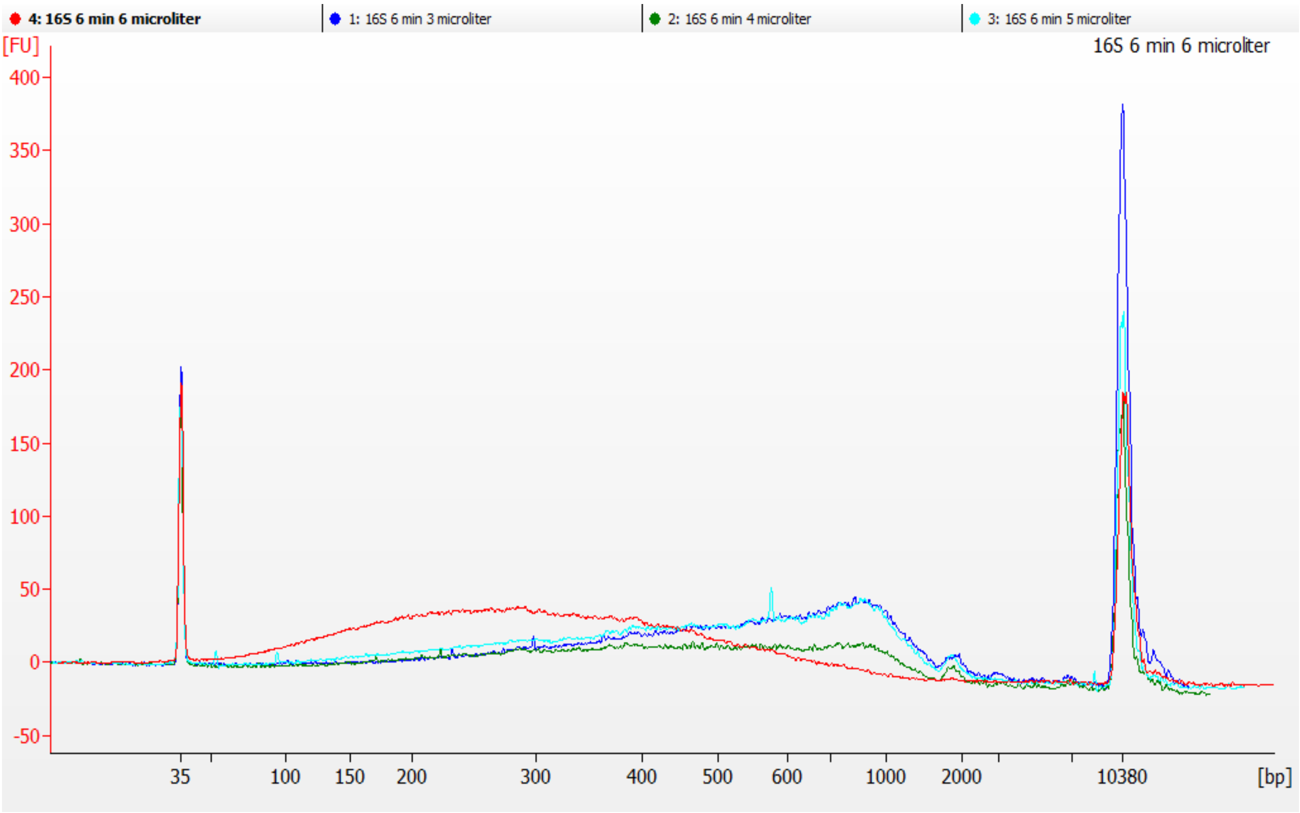


| | From [bp] | To [bp] | % of Total | Average Size [bp] | Conc. [pg/µl] | Molarity [pmol/l] |
|---|---|---|---|---|---|---|
| 16S 6 min 3 microliter | 75 | 8,000 | 97 | 678 | 428.47 | 1,645.80 |
| 16S 6 min 4 microliter | 75 | 8,000 | 97 | 654 | 679.77 | 3,039.20 |
| 16S 6 min 5 microliter | 75 | 8,000 | 98 | 603 | 822.4 | 3,634.90 |
| 16S 6 min 6 microliter | 75 | 8,000 | 96 | 324 | 1,182.02 | 7,974.90 |

| **DNA (ng) in total shearing solution** | |
|---|---|
| **Bioanalzyer** (conc*50) | **Qubit** (10ng/microl * volume) |
| 21.4 | 30.0 |
| 34.0 | 40.0 |
| 41.1 | 50.0 |
| 59.1 | 60.0 |

Pre-shearing for Spiked-DNA-GP: Pre-shearing for Spiked-DNA-SP: 30 and 40 ng was used to test the effect of shearing. Shearing of 40 ng had the best shearing and given the fact that there was only one try with the actual sample, the safer option of 40 ng  was opted.
Too little DNA could result in a graph too close to the baseline to distinguish the sample from said baseline. (top graph and table)

Bottom table: The amounts of DNA according to Qubit and Bioanalyzer measurements. The Qubit measurement of 16S ribosomal amplicon was 10 ng/microliter. For the Qubit, this was multiplied by the amount of microliters used i.e. (10 ng/microl * volume). The Bioanalyzer samples were dissolved in 50 microliters, which was multiplied by the concentration in pg/microliter (conc*50).

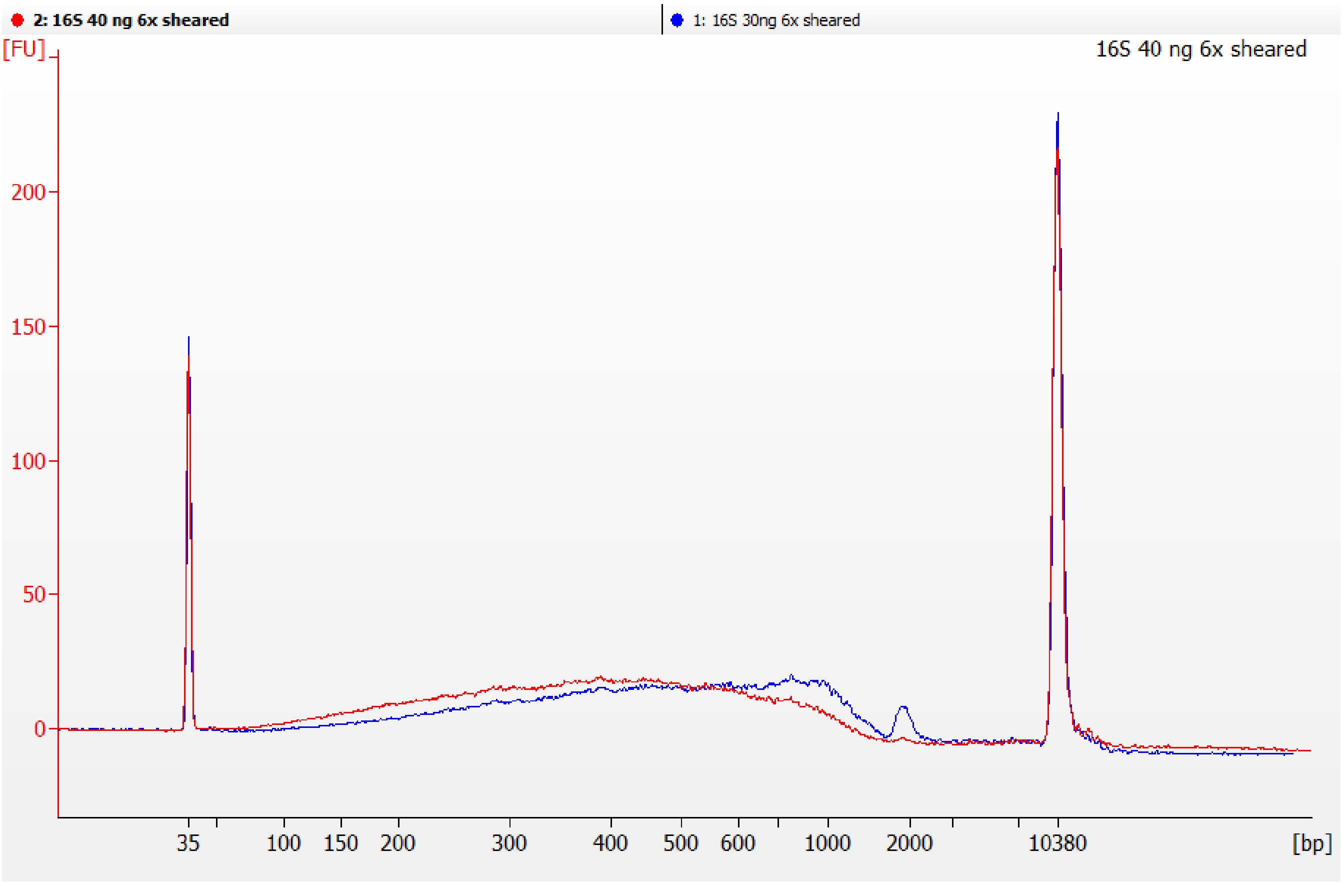


| | From [bp] | To [bp] | % of Total | Average Size [bp] | Conc. [pg/µl] | Molarity [pmol/l] |
|---|---|---|---|---|---|---|
| 16S 30 ng 6x | 75 | 8,000 | 97 | 706 | 538.04 | 2,306.60 |
| 16S 40 ng 6x | 75 | 8,000 | 96 | 495 | 651.14 | 3,455.60 |

| **DNA (ng) in total shearing solution** | |
|---|---|
| **Bioanalzyer** (conc*50) | **Qubit** (10ng/ml * volume) |
| 26.9 | 30.0 |
| 32.5 | 40.0 |

Pre-shearing for MDA: Three shearing times were tested with 4 to 7 concentrations in order to determine the lower limits of the Bioanalyzer. From this, it appeared that quantities of DNA lower than 40 ng could approach baseline levels and are therefore not suitable for further steps.

Bottom table: The amounts of DNA according to Qubit and Bioanalyzer measurements. The Qubit measurement of MDA amplified DNA was 10,8 ng/microliter. For the Qubit, this was multiplied by the amount of microliters used i.e. (10 ng/microl * volume). The Bioanalyzer samples were dissolved in 50 microliters, which was multiplied by the concentration in pg/microliter (conc*50).

**4 minutes shearing time**

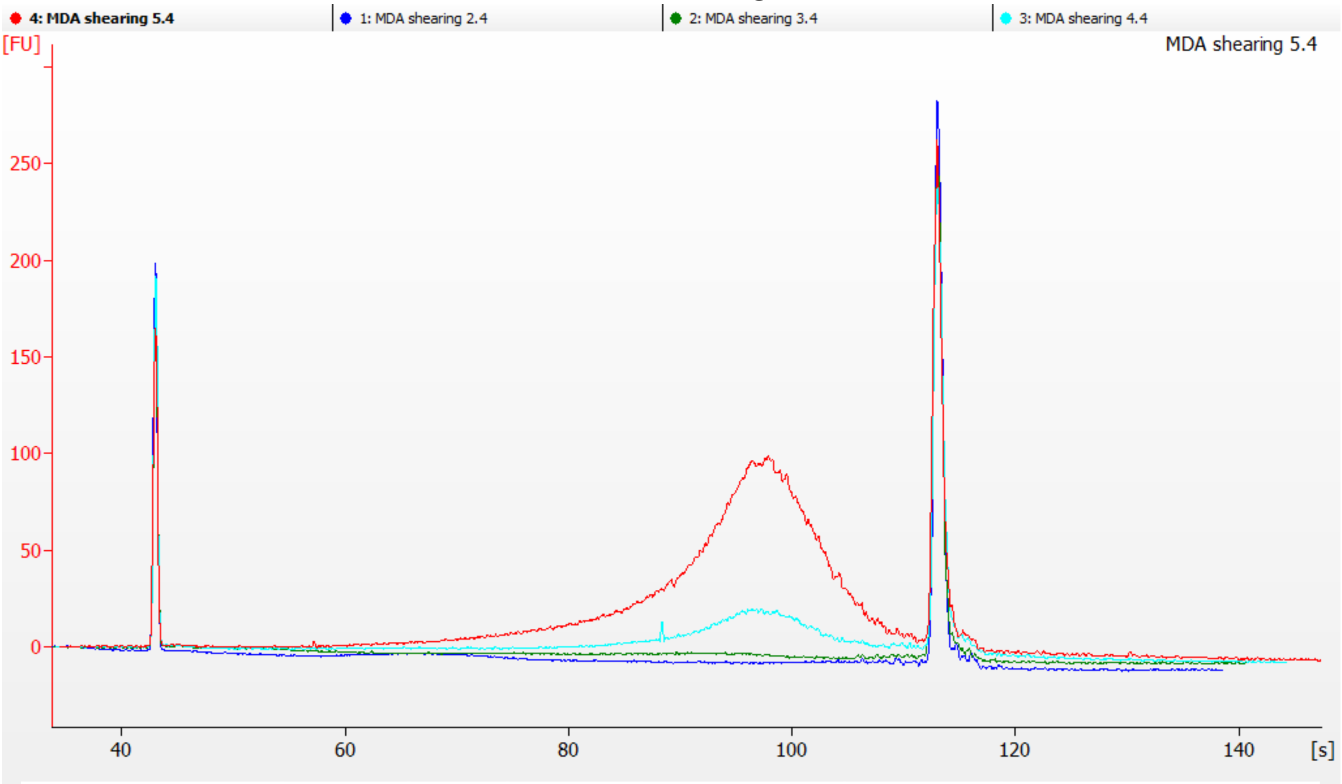


| | From [bp] | To [bp] | % of Total | Average Size [bp] | Conc. [pg/µl] | Molarity [pmol/l] |
|---|---|---|---|---|---|---|
| MDA 2.4 (21.6 ng) | 75 | 8,000 | 28 | 4,980 | 1.78 | 2.5 |
| MDA 3.4 (32.4 ng) | 75 | 8,000 | 67 | 2,085 | 10.98 | 58.7 |
| MDA 4.4 (43.2 ng) | 75 | 8,000 | 91 | 1,581 | 171.74 | 440.7 |
| MDA 5.4 (54.0 ng) | 75 | 8,000 | 96 | 1,483 | 625.94 | 1,327.80 |

| DNA (ng) in total shearing solution | |
|---|---|
| **Bioanalzyer** (conc*50) | **Qubit** (10,8 ng/ml * volume) |
| 0.09 | 21.6 |
| 0.55 | 32.4 |
| 8.87 | 43.2 |
| 31.3 | 54.0 |

Pre-shearing for MDA: Three shearing times were tested with 4 to 7 concentrations in order to determine the lower limits of the Bioanalyzer. From this, it appeared that quantities of DNA lower than 40 ng could approach baseline levels and are therefore not suitable for further steps.

Bottom table: The amounts of DNA according to Qubit and Bioanalyzer measurements. The Qubit measurement of MDA amplified DNA was 10,8 ng/microliter. For the Qubit, this was multiplied by the amount of microliters used i.e. (10 ng/microl * volume). The Bioanalyzer samples were dissolved in 50 microliters, which was multiplied by the concentration in pg/microliter (conc*50).

**6 minutes shearing time**

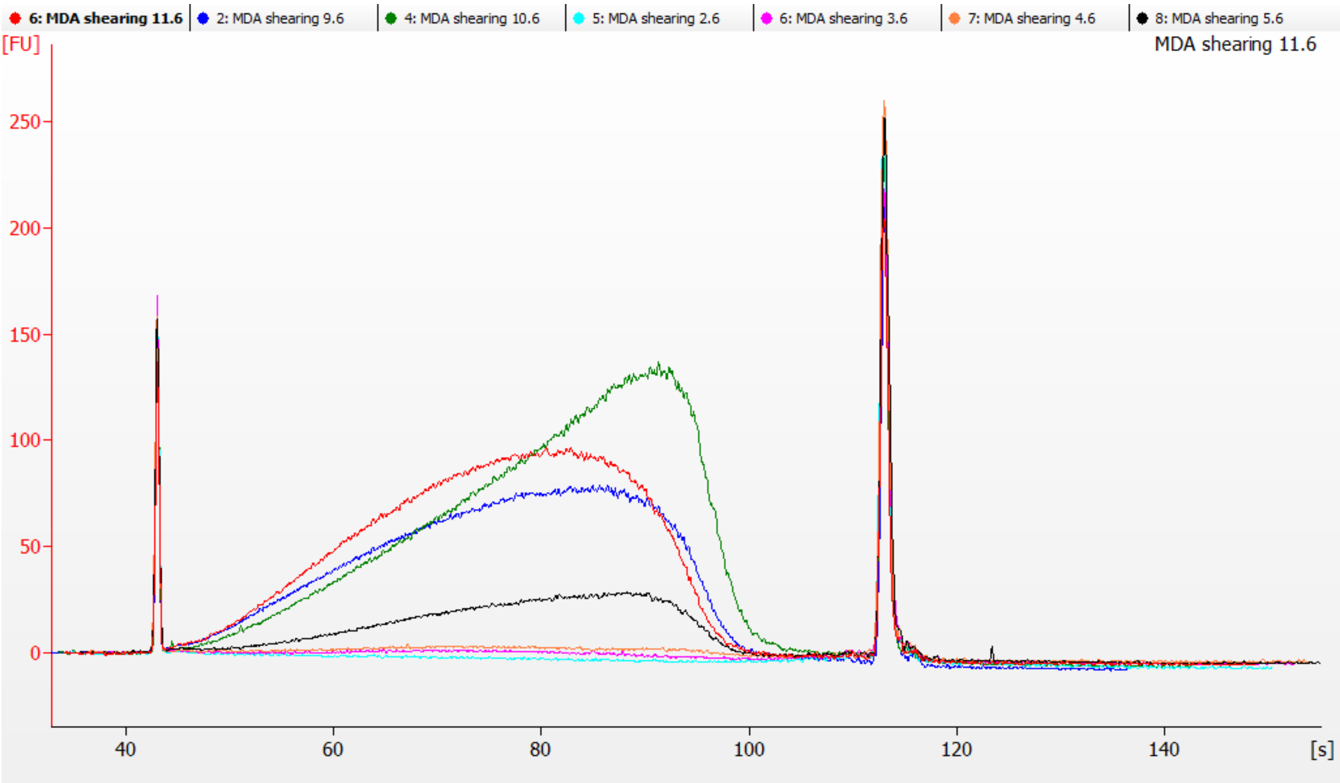


| | From [bp] | To [bp] | Corr. Area | Average Size [bp] | Conc. [pg/µl] | Molarity [pmol/l] |
|---|---|---|---|---|---|---|
| MDA 2.6 (21.6 ng) | 75 | 8,000 | 37.4 | 3,148 | 18.62 | 131.3 |
| MDA 3.6 (32.4 ng) | 75 | 8,000 | 109.8 | 1,191 | 59.92 | 320.5 |
| MDA 4.6 (43.2 ng) | 75 | 8,000 | 233.2 | 880 | 117.03 | 639.1 |
| MDA 5.6 (54.0 ng) | 75 | 8,000 | 1,158.40 | 580 | 581.37 | 2,699.30 |
| MDA 9.6 (97.2 ng) | 75 | 8,000 | 3,810.40 | 510 | 2,387.84 | 12,435.80 |
| MDA 10.6 (108 ng) | 75 | 8,000 | 4,749.30 | 612 | 2,675.53 | 11,634.60 |
| MDA 11.6 (118.8 ng) | 75 | 8,000 | 4,266.80 | 463 | 2,764.75 | 14,475.80 |

| DNA (ng) in total shearing solution | |
|---|---|
| **Bioanalzyer** (conc*50) | **Qubit** (10,8 ng/ml * volume) |
| 0.9 | 21.6 |
| 3 | 32.4 |
| 5.9 | 43.2 |
| 29.1 | 54.0 |
| 119.3 | 97.2 |
| 133.8 | 108.0 |
| 138.2 | 118.8 |

Pre-shearing for MDA: Three shearing times were tested with 4 to 7 concentrations in order to determine the lower limits of the Bioanalyzer. From this, it appeared that quantities of DNA lower than 40 ng could approach baseline levels and are therefore not suitable for further steps.

Bottom table: The amounts of DNA according to Qubit and Bioanalyzer measurements. The Qubit measurement of MDA amplified DNA was 10,8 ng/microliter. For the Qubit, this was multiplied by the amount of microliters used i.e. (10 ng/microl * volume). The Bioanalyzer samples were dissolved in 50 microliters, which was multiplied by the concentration in pg/microliter (conc*50).

**8 minutes shearing time**

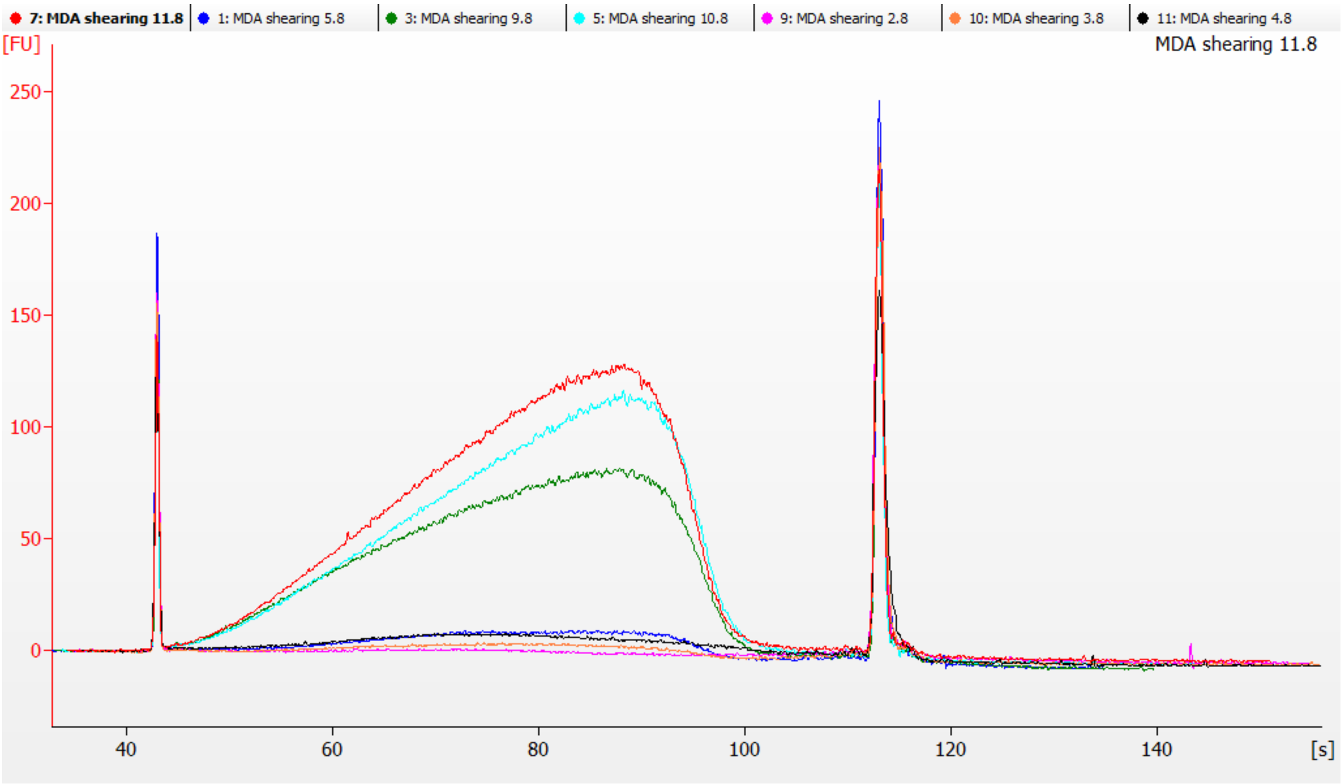


| | From [bp] | To [bp] | Corr. Area | Average Size [bp] | Conc. [pg/µl] | Molarity [pmol/l] |
|---|---|---|---|---|---|---|
| MDA 2.8 (21.6 ng) | 75 | 8,000 | 110.5 | 1,495 | 60.96 | 302.7 |
| MDA 3.8 (32.4 ng) | 75 | 8,000 | 209.1 | 739 | 112.01 | 530.1 |
| MDA 4.8 (43.2 ng) | 75 | 8,000 | 477.1 | 787 | 287.73 | 1,489.60 |
| MDA 5.8 (54.0 ng) | 75 | 8,000 | 590.5 | 710 | 289.79 | 1,411.90 |
| MDA 9.8 (97.2 ng) | 75 | 8,000 | 3,732.90 | 542 | 2,124.21 | 10,631.70 |
| MDA 10.8 (108 ng) | 75 | 8,000 | 4,408.60 | 550 | 2,855.04 | 13,153.20 |
| MDA 11.8 (118.8 ng) | 75 | 8,000 | 4,933.00 | 533 | 2,898.54 | 13,630.20 |

| DNA (ng) in total shearing solution | |
|---|---|
| **Bioanalzyer** (conc*50) | **Qubit** (10,8 ng/ml * volume) |
| 3 | 21.6 |
| 5.6 | 32.4 |
| 14.3 | 43.2 |
| 14.5 | 54.0 |
| 106.2 | 97.2 |
| 142.8 | 108.0 |
| 145 | 118.8 |
